# Supplementary figures and images for: Text mining-based word representations for biomedical data analysis and protein-protein interaction networks in machine learning tasks
Source: PLoS One. 2021 Oct 15;16(10):e0258623. doi: 10.1371/journal.pone.0258623 (PMC8519453; doi:10.1371/journal.pone.0258623)

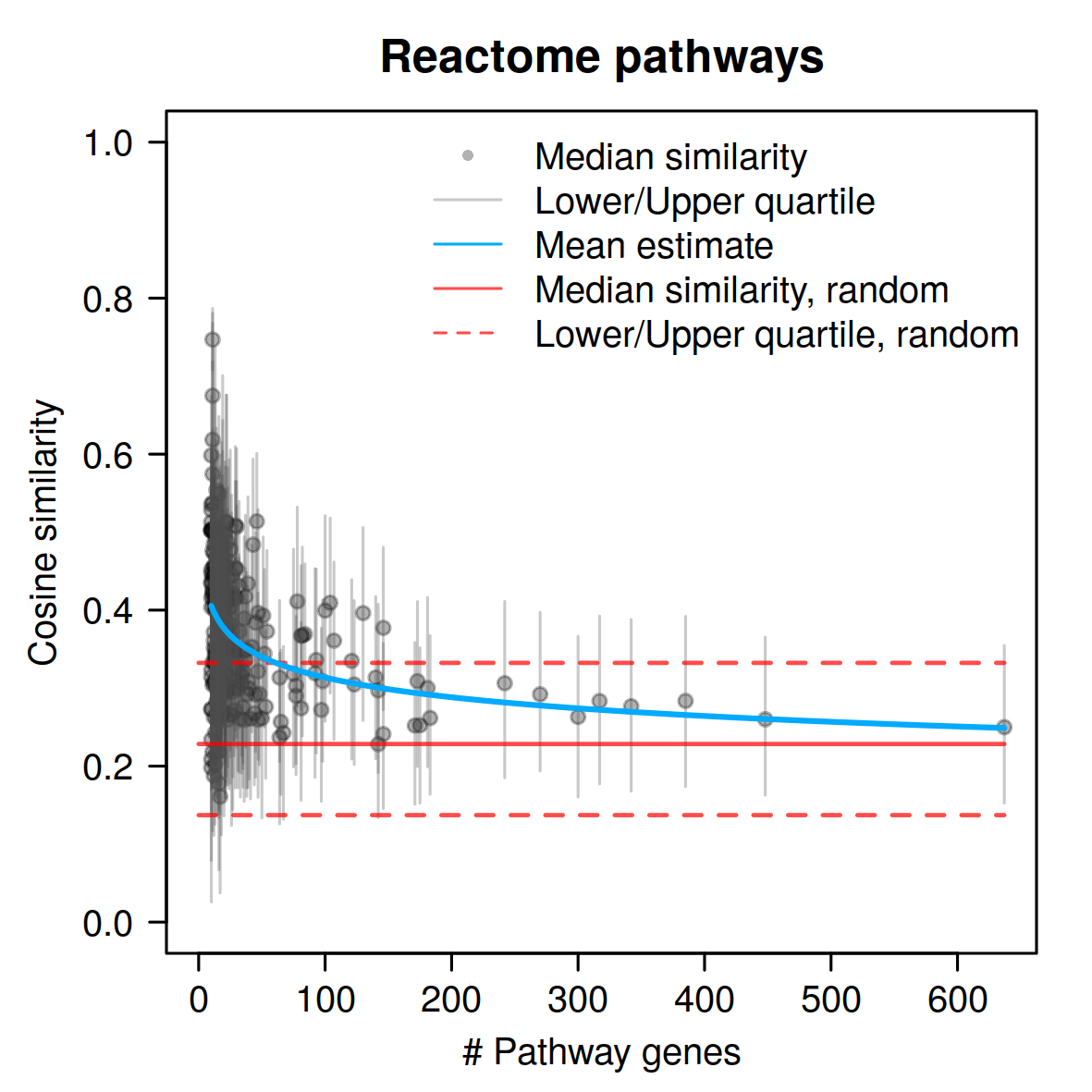

Supplement: S1 Fig — Median, lower and upper quartiles are presented for a random sample of 2000 gene pairs. A mean trend was estimated using the function f(x) = (xa + b)−1. (TIF) [file pone.0258623.s001.tif]

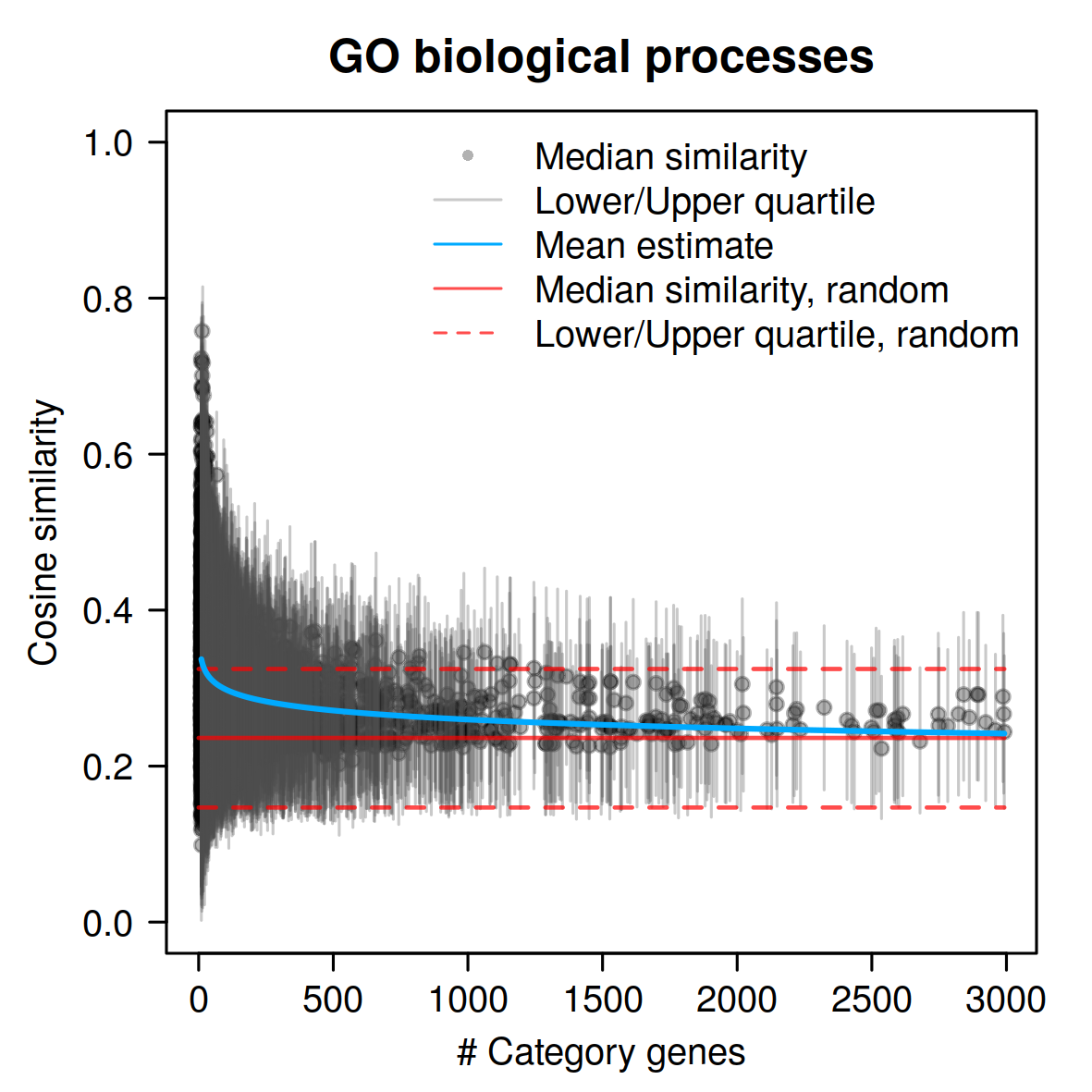

Supplement: S2 Fig — Median, lower and upper quartiles are presented for a random sample of 2000 gene pairs. A mean trend was estimated using the function f(x) = (xa + b)−1. (TIF) [file pone.0258623.s002.tif]

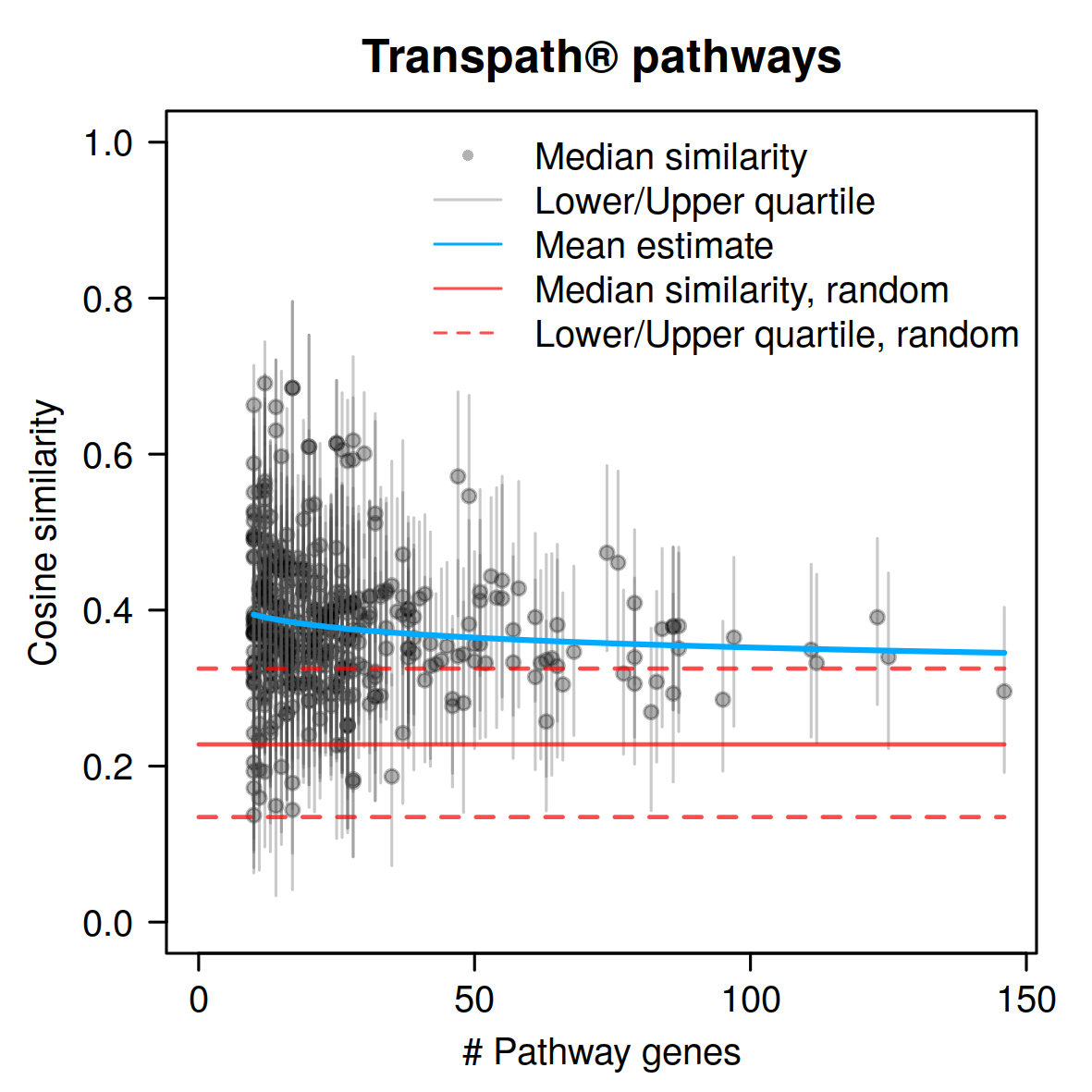

Supplement: S3 Fig — Median, lower and upper quartiles are presented for a random sample of 2000 gene pairs. A mean trend was estimated using the function f(x) = (xa + b)−1. (TIF) [file pone.0258623.s003.tif]

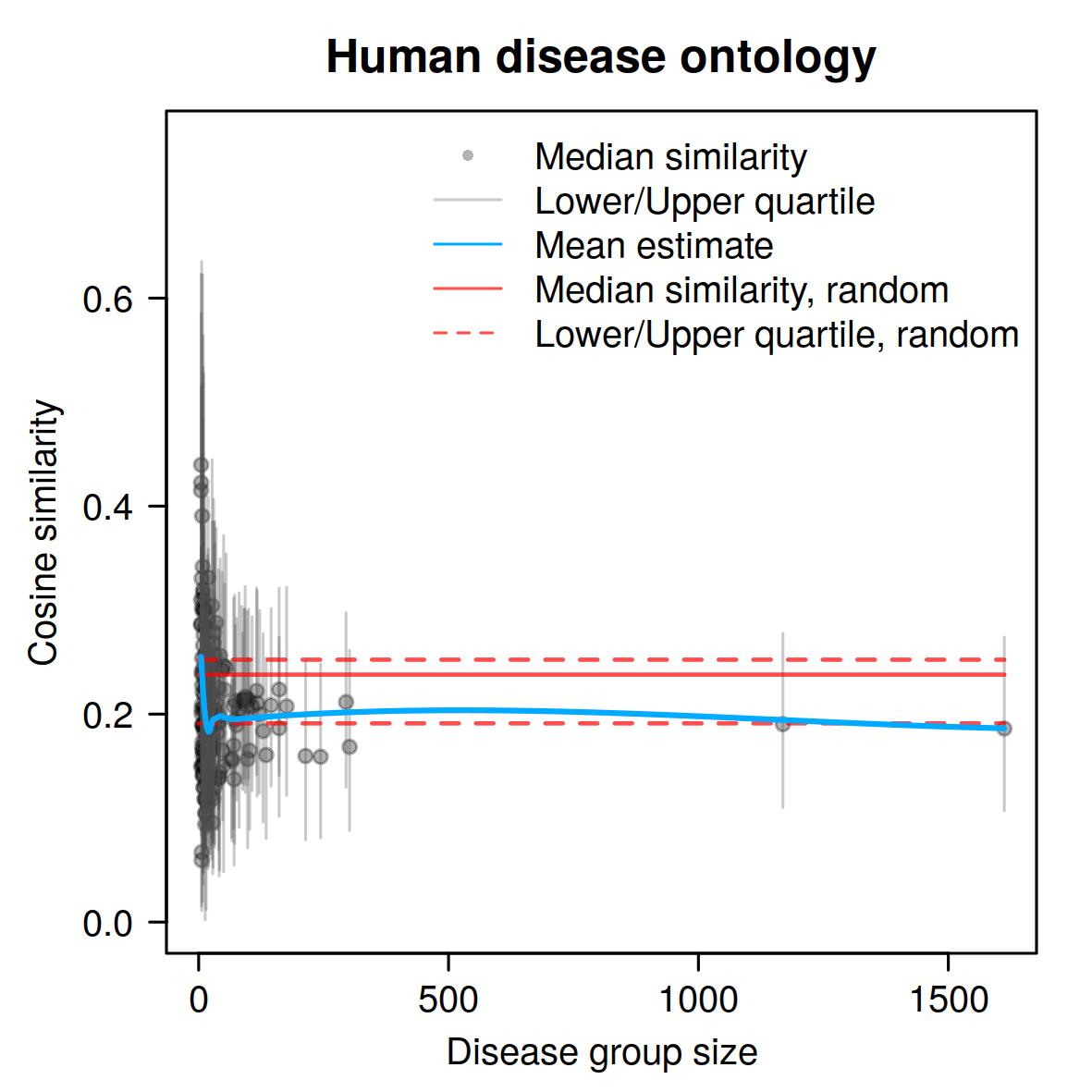

Supplement: S4 Fig — Median, lower and upper quartiles are presented for a random sample of 700 disease pairs. A mean trend was estimated by non-parametric local regression (Loess). (TIF) [file pone.0258623.s004.tif]

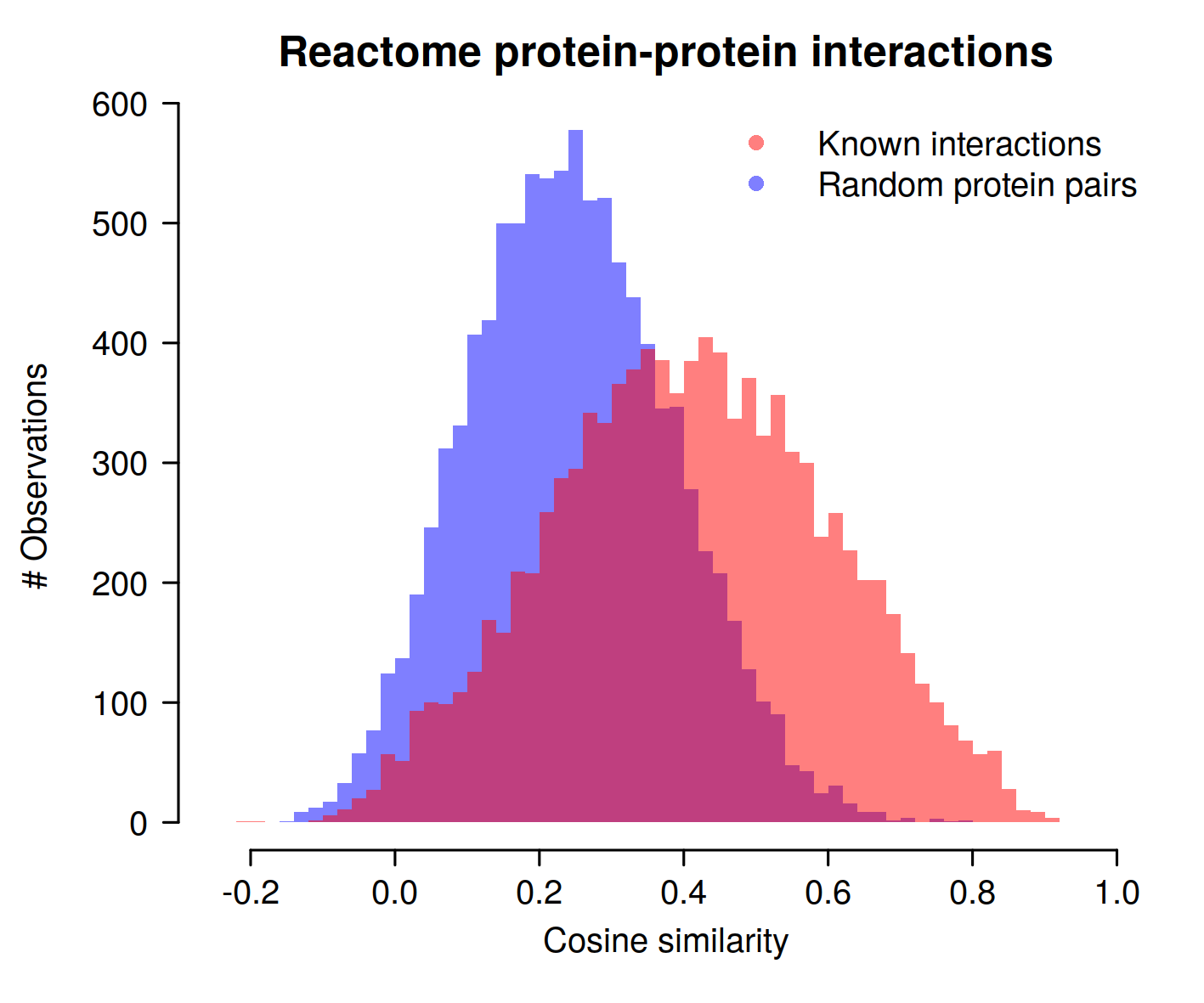

Supplement: S5 Fig — Each group contained a random sample of 10000 pairs. (TIF) [file pone.0258623.s005.tif]

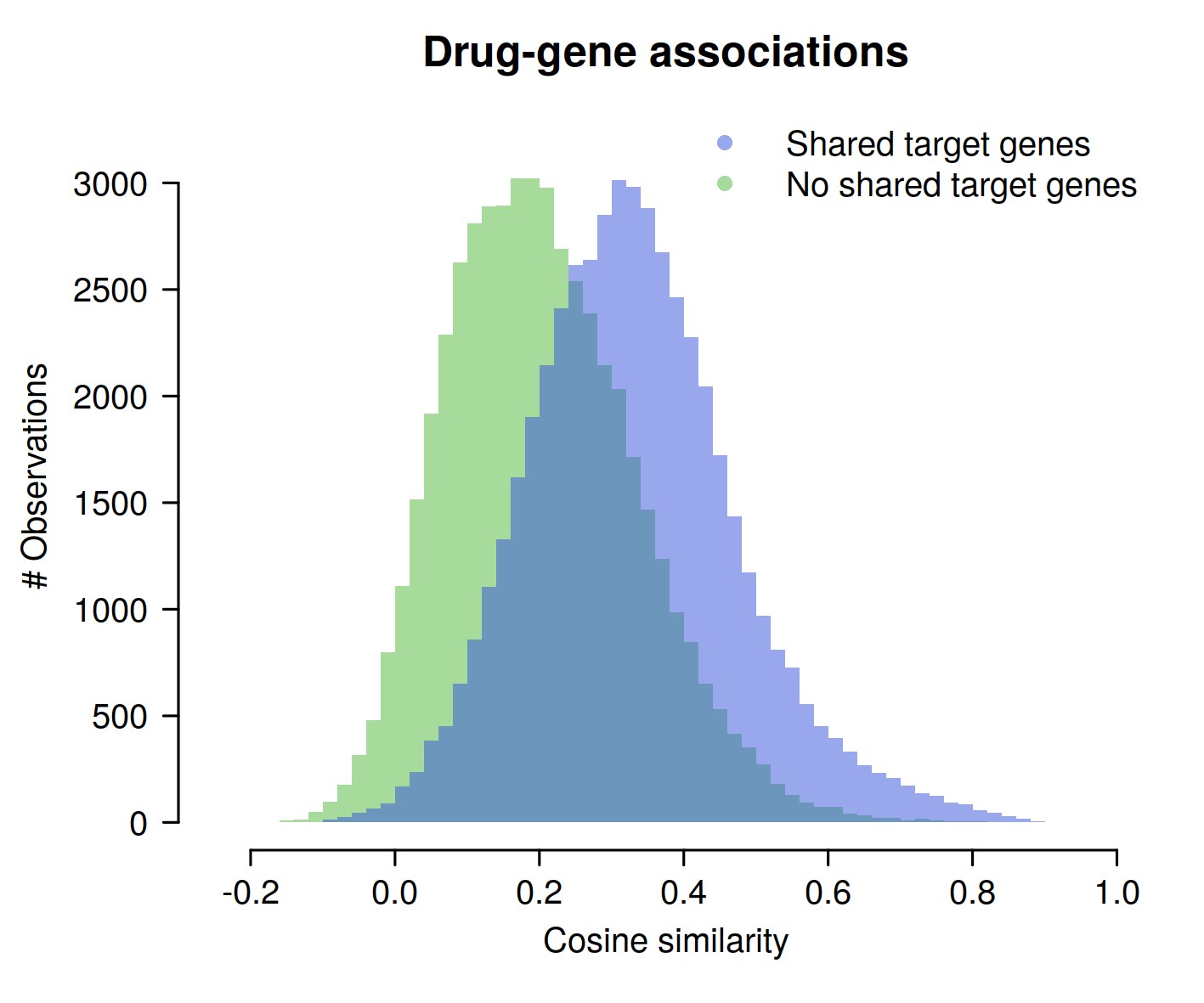

Supplement: S6 Fig — Each group contained a random sample of 50000 drug pairs. (TIF) [file pone.0258623.s006.tif]

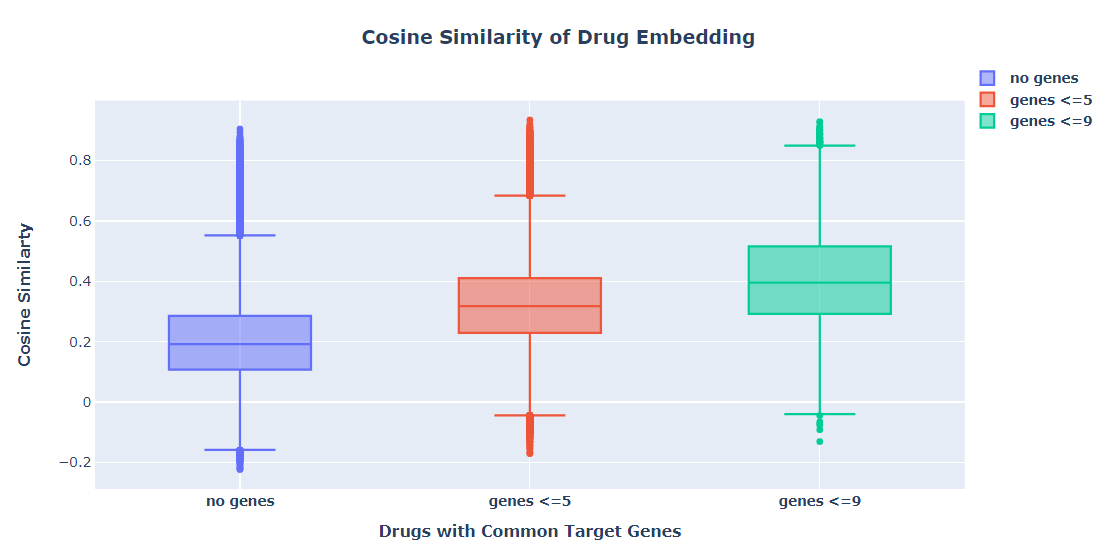

Supplement: S7 Fig — Drug-drug groups were estimated by counting the number of shared genes between two drugs presented in the embedding. Group1 (no genes: median = 0.192, lower quartile = 0.108, upper quartile = 0,286), group2 (genes ≤ 5: median = 0.318, lower quartile = 0.229, upper quartile = 0,411), group3 (genes ≤ 9: median = 0.396, lower quartile = 0.292, upper quartile = 0,516). (TIF) [file pone.0258623.s007.tif]

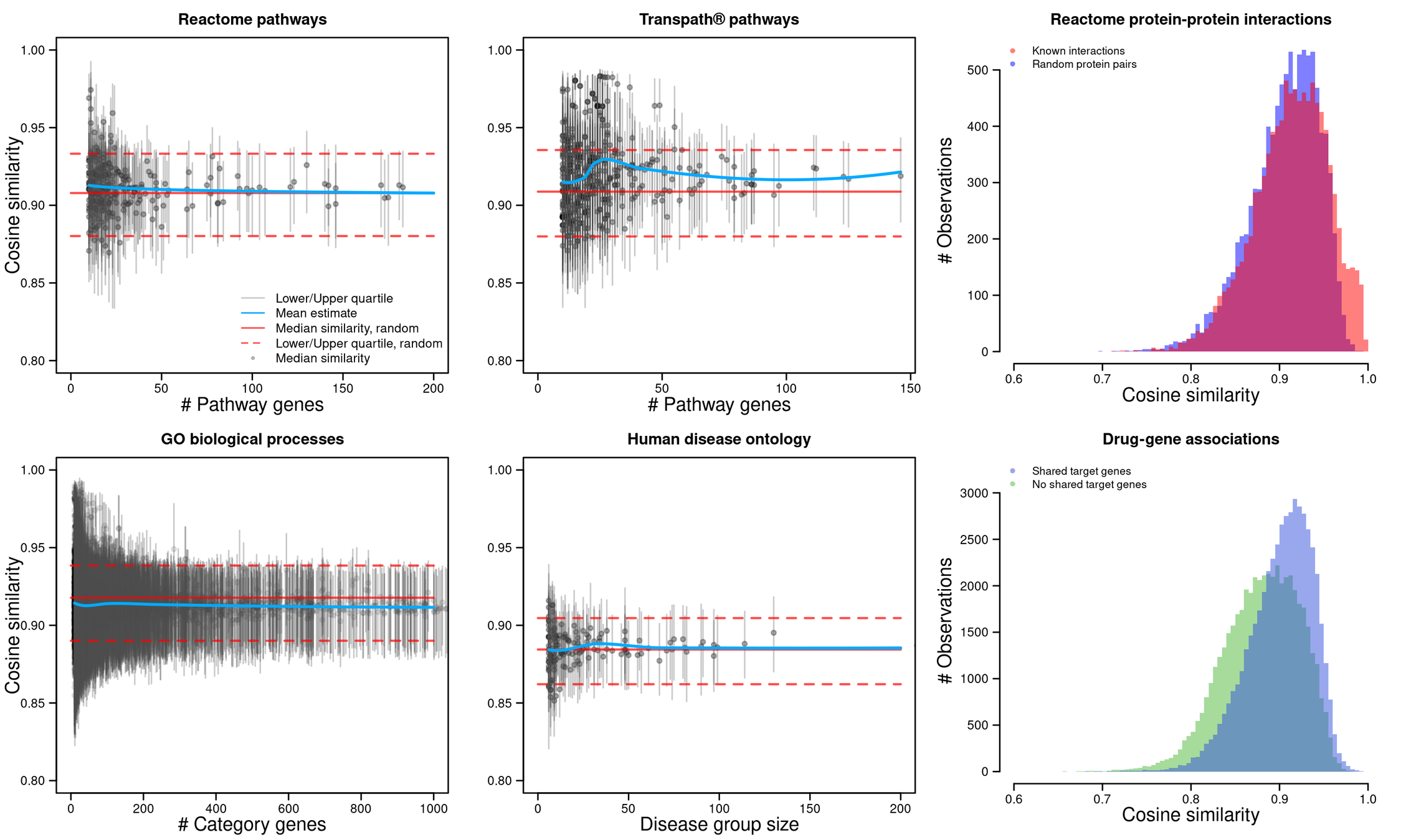

Supplement: S8 Fig — (TIF) [file pone.0258623.s008.tif]
